# Valorization of Legume By-Products Based on Polyphenols and Protein Contents for Potential Nutraceutical Applications

**DOI:** 10.3390/antiox13121531

**Published:** 2024-12-14

**Authors:** Cristina Terenzi, Gabriela Bermudez, Francesca Medri, Serena Montanari, Franz Bucar, Vincenza Andrisano

**Affiliations:** 1 Department for Life Quality Studies, University of Bologna, Corso D’Augusto 237, 47921 Rimini, Italy; cristina.terenzi2@unibo.it (C.T.); gabriela.bermudez2@unibo.it (G.B.); francesca.medri4@unibo.it (F.M.); serena.montanari5@unibo.it (S.M.); 2 Institute of Pharmaceutical Sciences, Department of Pharmacognosy, University of Graz, Beethovenstraße 8, 8010 Graz, Austria; franz.bucar@uni-graz.at

**Keywords:** legume by-products, polyphenols, UHPLC-DAD-ESI-MS^n^, antioxidant activity, protein content, pesticide-free

## Abstract

A significant amount of agri-food by-products is generated by large food industry production lines. Aligned with the principles of a circular economy, this project aims to recycle and valorize legumes, such as beans, green beans and soy by-products characterized by different heat treatments, maturation stages and cultivation methods. The valorization of food waste involved the development of an Ultrasound-Assisted Extraction (UAE) method to isolate polyphenols. Analytical techniques, including UHPLC-DAD-ESI-MS^n^, were used to identify polyphenols in legume, green bean and soy extracts obtained through UAE. Additionally, UV-Vis spectrophotometric assays measured the Total Phenolic Content (TPC) and Total Antioxidant Status (TAS), while the Kjeldahl method was employed to assess the protein content in each UAE extract. The analyses revealed a variety of valuable polyphenols in legume, green bean and soy by-products. For instance, bean by-products contain feruloyl glucaric acid derivatives, green beans by-products have different types of flavonols such as quercetin-3-O-glucuronide, and soy by-products are rich in isoflavones. These findings demonstrate the potential for formulating nutraceuticals from these by-products’ extracts.

## 1. Introduction

Nowadays, food industries are facing a significant challenge related to sustainability, largely due to the problem of food waste. This issue has serious implications for both the economy and the environment. It has been found that about 14% of global food is lost between the point of harvesting and reaching the point of sale, and another 17% is wasted by consumers [1]. As a result, the annual global volume of food waste is estimated at 1.3 billion tons [2]. Given these numbers and the inefficiencies in managing food waste, it can be argued that waste reduction is a major challenge. One of the proposed strategies is the transition from the traditional linear economy model to the circular economy model [3]. In the linear economy, the process starts with raw materials and ends in waste, while the circular economy model considers waste as valuable resources that can be used in the economic cycle. This approach allows for the optimal conservation of resources by reusing, recycling, and recovering materials for as long as possible, aiming to extend their life cycle and minimize waste production [3].

In the global food industry, horticulture is the leading sector in terms of production, accounting for 38% of agricultural production and 65% of plant production [4]. However, large-scale horticultural industries produce a significant amount of waste, most of which is incinerated in landfills, releasing polluting gases [5].

Agri-food waste, often discarded as a by-product of food production and consumption, is increasingly recognized as a valuable source of bioactive compounds and essential nutrients [6,7,8]. Numerous studies have demonstrated the potential of these waste materials to contribute to sustainable food systems and human health. The literature provides many examples showing that agri-food waste is still rich in active metabolites and key nutrients [4,9].

Building on these premises, this study focuses on the valorization of selected by-products and wastes from an agri-food consortium in the Romagna region of Italy, aiming to determine whether they are still as rich in bioactive compounds such as polyphenols and proteins as the final product.

The agri-food consortium provided samples of both beans and green beans (*Phaseolus vulgaris* L.) from the final production of frozen legumes. The bean samples included both by-products (peels, leaves, hulls) and the final product for sale, while the green bean samples consisted only of waste. Additionally, soy samples were provided, specifically the “soy raw material” refers to seeds used as the initial source in soy milk production while the “legume by-product”, called okara, denotes the waste material generated during the industrial processing stage.

In this study, in the case of the bean samples, a comparison was made between the final product, intended for sale, and the generated waste material, while for the soy samples, a comparison was performed between the initial raw material, the seeds, and the by-product, okara. Conversely, for the green bean samples, the comparison was performed directly between by-products, as the corresponding final product was not supplied. Samples were also collected from different steps of the production chain (pre- and post-cooking). Moreover, two distinct maturation stages were observed for the *Phaseolus vulgaris* L. species: green beans (young stage; fruits and seeds) and beans (more mature stage; seeds).

Furthermore, all samples were cultivated using three different approaches: biological, conventional, and “Lotta Integrate” (LI). These methods vary in terms of the types and quantities of pesticides employed. Conventional agriculture is an intensive farming system maximizing land use and yield to meet global food demands. However, this approach is often based on heavily chemical pesticide use and fertilization, leading to significant environmental alarms and high energy consumption [10]. In contrast, biological farming, regulated by EU Regulation 2018/848, emerges as a sustainable agricultural alternative. This method emphasizes the use of natural substances and processes, exploiting the soil fertility and respecting natural cycles. While biological systems may yield less compared to conventional methods, they generally protect the environment [10]. LI offers another approach to agricultural production, representing a middle way between conventional and biological farming, aiming to minimize chemical use and reduce environmental impact. This strategy balances economic and production needs with environmental health considerations [11].

Therefore, to explore the potential for secondary use in health, the valorization of by-products involved determining their content of bioactive molecules, polyphenols, and proteins. This analysis was performed in comparison to the respective final products, as in the case of beans and green beans, which belong to the same species, *Phaseolus vulgaris* L. The comparison between young and old stages, as well as green beans and beans, proved to be particularly interesting. Additionally, the comparison of okara with its direct “raw material”, soy seeds, was of significant curiosity. From a valorization and originality standpoint, a notable aspect was the comparison between different cultivation methods mentioned above (biological, conventional, and LI). This analysis highlighted the influence of cultivation practices on the bioactive molecule content, particularly polyphenols, in various agri-food products and waste.

Thus, the valorization of food waste and by-products was achieved through the development of an extraction method based on Ultrasound-Assisted Extraction (UAE), which was used to isolate polyphenols.

Furthermore, the extracts were characterized by a UHPLC-DAD-ESI-MS^n^ (Ultra-High Performance Liquid Chromatography-Diode Array Detector-Electrospray Ionization-Mass Spectrometry) chromatographic method and two distinct UV-Vis spectrophotometric assays: Total Phenolic Content (TPC) to determine the polyphenolic content and Total Antioxidant Status (TAS) to evaluate the antioxidant activity of the extracts. Finally, the Kjeldahl method was applied to determine the protein content of each sample.

## 2. Materials and Methods

### 2.1. Chemicals and Reagents

Methanol for HPLC-MS, Water for HPLC-MS, Acetic acid for HPLC-MS, Methanol HPLC grade ≥ 99.9%, methanol ≥ 99.8%, Acetone HPLC grade ≥ 99.8%, Ethanol and acetic acid ≥ 99.8%, Potassium persulfate ≥ 99.0%, ABTS (2,2-azinobis-(3ethylbenzothiazoline-6-sulfonate)), Trolox^®^ (6-hydroxy-2,5,7,8-tetramethyl-3,4-dihydrochro mene-2-carboxylic acid), Sodium hydroxide ≥ 98% in pellets, Sodium carbonate ≥ 99.5% were purchased from Sigma-Aldrich (Taufkir-chen, Germany). Folin–Ciocalteu reagent were purchased from VWR Chemicals (Darmstadt, Germany). Copper (II) sulfate pentahydrate and Potassium sodium tartrate tetrahydrate EMSURE^®^ were purchased from Merck (Darmstadt, Germany). Gallic acid, Protocatechuic acid, Trans-cinnamic acid, Caffeic acid ≥ 98.0%, p-coumaric acid ≥ 98.0%, Ferulic acid, (+)-Catechin, Isoquercitrin, Daidzein, Genistein, and Phloridzin dihydrate were purchased from Sigma-Aldrich (Taufkirchen, Germany). Chlorogenic acid, ≥98.0% was purchased from Apollo Scientific (Bredbury, UK). Quercitrin was purchased from Cayman Chemical (Ann Arbor, MI, USA). (−)-Epicatechin > 97.0%, Hesperetin > 97.0% and Apigenin > 97.0% were purchased from TCI (Zwijndrecht, Belgium). Hyperoside was purchased from HWI group (Rulzheim, Germany). (+)-Rutin trihydrate 97% was purchased from Alfa Aesar (Haverhill, MA, USA). Myricetin 98%, Naringenin 97% and Kaempferol ≥ 98.0% were purchased from ThermoFisher (Kandel, Germany). Naringenin chalcone was purchased from PhytoLab (Vestenbergsgreuth, Germany).

### 2.2. Equipment

Dionex UltiMate 3000 RS system including a Diode Array Detector (DAD) coupled to a LTQ XL^TM^ linear ion-trap mass spectrometer-ESI ion source from Thermo Fisher Scientific (Waltham, MA, USA). The Thermo XCalibur software, version 3.1.66.10 (Waltham, MA, USA), was employed for the acquisition of mass chromatograms and spectral data. Ultrasound Bath from Bandelin Sonorex (Berlin, Germany); Analytical balance from Sartorius; Centrifuge from Heraeus Biofuge Pico (Barcellona, Spain); Spectrophotometer: Jasco UV-VIS V-630 (Jasco Europe, Lecco, Italy). Ultrasound Bath: Elmasonic S 40 H (GEASS S.R.L., Turin, Italy). Centrifuge: Awel International MF 20-R multifunction centrifuge (MedWrench, East Point, GA, USA). Freeze-dryer: Alpha 1–4 LO plus (Martin Christ, Harz, Germany). Rotatory evaporator: IKA Rotary Evaporators RV 10 basic (IKA-Werke GmbH & Co. KG, Staufen im Breisgau, Germany).

### 2.3. Samples

Beans, green beans, and soy matrices were provided by Fruttagel S.C.p.A (Alfonsine, Italy). The samples were supplied already freeze-dried. The materials were then ground in two steps. First, a domestic mixer was used to break the solid materials and reduce particle size. Next, a second milling process (using an IKA Tube Mill 100 control) was employed to obtain fine powders. These powders were then cryo-lyophilized for 24 h at −60 °C and stored at −20 °C until use.

Legume by-products (ByP), final products (FinalP), and raw material (RM) are reported in Table 1. In this context, in the case of soy, the term “raw material” refers to the seeds that are used as the initial source material for the production of soy milk. In contrast, the by-product is the waste material obtained during the industrial processing stage.

Lotta Integrate (LI)* is a crop defense practice that aims to reduce pesticide use through the implementation of multiple measures. It is the intermedium between conventional and biological methodologies.

The products were classified as either biological or conventional based on the following criteria. Conventional products are obtained using active compounds regulated by Reg. (CE) No 396/2005. In contrast, biological products are obtained through cultivation practices governed by Reg. (CE) 848/2018. The primary objective of biological methodologies is to minimize chemical treatments by employing microorganisms or insects to naturally combat harmful organisms. In this case, the chemicals used are carefully selected based on their environmental impact. All samples analyzed were certified pesticide-free by the supplier, Fruttagel, using the 2018 UNI EN 15662 national procedure [12].

### 2.4. Ultrasound-Assisted Extraction (UAE) of Polyphenols from Agri-Food Samples

About 0.5 g of each lyophilized sample were weighed (n = 3), to which 4 mL of a mixture 50:50 methanol:water (containing 3.4% acetic acid) (Solution A) was added. The suspension was vortexed for 1 min and subjected to an ultrasonic bath (Elmasonic S 40 H) at 25 °C for 15 min. The solution was centrifuged at 3680× *g* (4400 rpm) at 20 °C for 15 min, and the supernatant collected in a 50 mL falcon tube. The procedure was repeated three times. The residual biomass was subjected to a second extraction step with 4 mL of a 70:30 acetone:water solution (Solution B), vortexed for 1 min, and subjected to UAE at 25 °C for 15 min. Then, the solution was centrifuged at 3680× *g* (4400 rpm) at 20 °C for 15 min and the supernatant placed in a 50 mL falcon tube. This extractive procedure was performed twice more. Once all 6 supernatants were collected, the last centrifugation at 3680× *g* (4400 rpm) at 20 °C for 5 min was performed, and the residual particles were removed. The obtained extracts were filtered through a nylon 0.45 μm filter, placed in a round bottom flask, and dried with a rotatory evaporator at 40 °C and with cryo-lyophilization for 24 h. Dried extracts were weighed for gravimetric determination and then stored in plastic Eppendorfs at −80 °C until use. The method has been already reported by Terenzi et al. [13].

### 2.5. Gravimetric Determination

Gravimetric determination was conducted on all dried extracts obtained with UAE to calculate the yield of extraction (%), as follows:

yield% = (grams of dried extract/grams of sample) × 100

Finally, to perform TPC assay, TAS assay, and UHPLC-DAD-ESI-MS^n^ analysis, the dried extracts were reconstituted with 25 mL of a mixture 50:50 Solution A:Solution B (*v*/*v*), described in Section 2.4, to obtain the sample solutions to test.

### 2.6. UHPLC-DAD-ESI-MS^n^ Analysis of Polyphenols

Polyphenolic compounds in legume UAE extracts were analyzed using a UHPLC-DAD-ESI-MS^n^ system. Chromatographic separation was achieved on a Kinetex C18 column (50 × 2.1 mm, 1.7 µm) kept at 20 °C. A gradient elution was employed, using a mobile phase consisting of 0.1% formic acid in water [A] and 100% methanol [B] at a flow rate of 0.250 mL/min. The gradient profile started with 2% [B] for 5 min, increasing to 10% [B] at 7.78 min, 30% [B] at 9.72 min, 37% [B] at 14 min, 60% [B] at 23.0 min, then dropping back to 2% [B] at 23.23 min and keeping this composition until the end (27 min). UV-Vis spectra were recorded on a DAD detector in the range of 190–500 nm, with signal acquisition at 280 nm.

Mass spectrometric analysis was performed using a linear ion trap mass spectrometer equipped with an electrospray ionization (ESI) source. Both positive and negative ion modes were applied. The ESI source parameters were set as follows: source voltage of 4.20 kV (positive) or 4.00 kV (negative), capillary temperature of 330 °C, source heater temperature of 250 °C, sheath gas flow of 50 arbitrary units, and auxiliary gas flow of 8 arbitrary units. Mass spectra were acquired over an m/z range of 50–2000.

Regarding sample preparation, all UAE extracts of 25 mL were subjected to a 10-times concentration and subsequent centrifugation at 13,000 rpm for 10 min before injection.

A semi-quantitative analysis was performed using DAD total scan chromatograms. Peak areas of standard reference compounds with known concentrations were compared to those of identified polyphenols in the UAE extracts to estimate their relative amount employing the following equation:

(1) $$Area\;Standard:Concentration\;Standard\left(\frac{µg}{\mathrm{mL}}\right)=Area\;Peak\;Sample:Concentration\;Peak\;Sample(\frac{µg}{\mathrm{mL}})$$

In the absence of a standard reference compound, semi-quantitative calculations were performed using the areas of polyphenol standards from the same class or family, as previously reported by Mesquita and Monteiro [14]. The quantity of polyphenols was expressed in milligrams per 100 g of dry weight (DW) of the lyophilized sample powder.

### 2.7. Total Phenolic Content Assay (TPC)

The phenolic content of the extracts was measured following the procedure described by Redmile-Gordon et al. [15]. The method involves the preparation of two reagents, Reagent A (reacts with proteins to subtract their interference) and Reagent B (not reactive with proteins; it allows for measuring polyphenols), composed of three different stock solutions: (1) 3.5 g of CuSO_4_·5H_2_O in 100 mL H_2_O, (2) 7 g of sodium potassium tartrate in 100 mL of H_2_O, and (3) 70 g of Na_2_CO_3_ in 1 L of NaOH 0.35 N solution. The three solutions were combined in sequence in the proportion 1:1:100 (*v*/*v*/*v*) to obtain Reagent A. Reagent B was prepared in the same way except for solution (1) being replaced by deionized water. The standard calibration curve was prepared with gallic acid as standard (MW: 170.12 g mol^−1^) to obtain solutions ranging from 15.71 to 31.43 μg mL^−1^. Standard and sample solutions (prepared as described in Section 2.4) were diluted 1:2 with deionized water. All the solutions were prepared in triplicate. Then, 400 μL of standard and sample dilutions were added to 400 μL of Reagent A. The same procedure was also performed for Reagent B. Samples and standards were incubated at room temperature in the dark for 10 min. After 10 min, 400 μL of Folin–Ciocalteu reagent 1:10, prepared immediately before the end of the first incubation (Folin 2N diluted 1:10 with deionized water and stored in the dark), were added to all standards and samples. Then, the reaction was carried out in the dark for 30 min at room temperature prior to measuring the absorbances at a wavelength of 750 nm (λmax). The blank was prepared by adding 400 μL of water, 400 μL of Reagent A or Reagent B (depending on the reagent used), and 400 μL of Folin–Ciocalteu reagent 1:10. The absorbance values measured at 750 nm (λmax) for Reagent A (AbsA) and Reagent B (AbsB) were used to calculate the absorbance of proteins and the absorbance of polyphenols following equations reported in the literature [15]:

Abs_proteins_ = 1.25 × (AbsA − AbsB)

Abs_polyphenols_ = AbsB − 0.2 × (Abs_proteins_)

The Abs_polyphenols_ was used to determine the TPC values, expressed as mg of gallic acid equivalents (GAE)/1 g of dry extract (Ext).

The method has been reported by Terenzi et al. [13].

### 2.8. Total Antioxidant Status (TAS)—ABTS^^•+^^ Radical Cation Scavenging Activity

TAS was determined through the Trolox Equivalent Antioxidant Capacity (TEAC) assay by spectrophotometric measurement of the ABTS^•+^ radical cation [16]. A solution of ABTS salt (7 mM) in water and a solution of potassium persulfate (2.45 mM) in water were prepared via sonication for 5 min. Then, the ABTS stock solution (A) and the potassium persulfate solution (B) were combined in a ratio of 2:1 (*v*/*v*) to generate ABTS^•+^ radical cation (C). ABTS oxidation was completed in 8 h via incubation in the dark at room temperature. Before measurement, the C solution was diluted 1:3 (*v*/*v*) in ethanol to obtain a working solution (D) with an absorbance of about 0.7 at 734 nm (λmax). A calibration curve was established using Trolox as a reference antioxidant, dissolving and diluting it in ethanol to obtain final concentrations in the range of 0.74–23.73 μM.

The D Solution, Trolox solutions, and samples (prepared in triplicate as described in Section 2.4) were equilibrated at 30 °C using the thermomixer. Then, the reaction was carried out directly in the cuvette, in the dark, by adding 10 μL of each Trolox solution and samples to 1.0 mL of solution D (Abs_734nm_ = 0.712), then mixing and waiting 1 min to measure the absorbances at 734 nm. Ethanol was used as a blank.

Once all absorbances at 734 nm were registered, the ∆Abs and the percentage of inhibition (I%) of ABTS^•+^ relative to each Trolox solution and sample were calculated as follows:

∆Abs_734nm_ = A − A1 (sample or Trolox solution)/A

and

I% = [(∆Abs_734nm_)/A] × 100;

A: control absorbance (ABTS^•+^);

A1: sample absorbance.

The TAS was expressed as µmol equivalent of Trolox (TE) 1 g^−1^ of dry extract (Ext). The method has been reported by Terenzi et al. [13].

### 2.9. Determination of the Protein Content by the Kjeldahl Method

The Kjeldahl method was conducted following the guidelines of the Association of Official Agricultural Chemists International (AOAC) [17]. About 1 g of agri-food sample powder lyophilized was hydrolyzed with 15 mL of concentrated sulfuric acid containing 2 copper catalysts at 420 °C for 2 h. After heating, H_2_O was added before proceeding with the neutralization and titration. The total nitrogen shared in the samples was multiplied by 6.25 as the conversion factor, as reported by the protocol. The method has been reported by Terenzi et al. [13].

### 2.10. Statistical Evaluation

All results were expressed as the mean ± SD of 3 independent experiments. Statistical analyses were performed using ordinary one-way ANOVA and Sidak’s multiple comparison tests. The statistical software GraphPad 10.0 version (GraphPad Prism, San Diego, CA, USA) was used, and *p*-values < 0.05 were considered statistically significant.

## 3. Result and Discussion

Different analytical methodologies were performed in this research to compare the by-products with the relative final products of legume UAE extracts. General tests such as gravimetric determination, TPC, TAS, and the Kjeldahl method were performed to compare extraction yields (%), polyphenolic content, antioxidant activity, and protein content. More selective and advanced techniques, such as UHPLC-DAD-ESI-MS^n^, were applied for the specific identification of the polyphenolic compounds in each UAE extract. Qualitative LC-MS analysis was also useful in highlighting differences between the young and mature stages of the *Phaseolus vulgaris* L. species.

### 3.1. Gravimetric Determination (Yield of Extraction)

The scientific literature suggests that bioactive compounds are frequently concentrated in the inner cellular structures of agricultural by-products [9]. Releasing these compounds often requires sophisticated extraction techniques. In recent times, unconventional extraction methods such as Ultrasound-Assisted Extraction (UAE), Microwave-Assisted Extraction (MAE), Supercritical Fluid Extraction (SFE), and others have increased in importance [9]. These methods offer advantages such as economic viability and safety, making them a better choice for extracting bioactive compounds from agricultural by-products.

**Figure 1:**
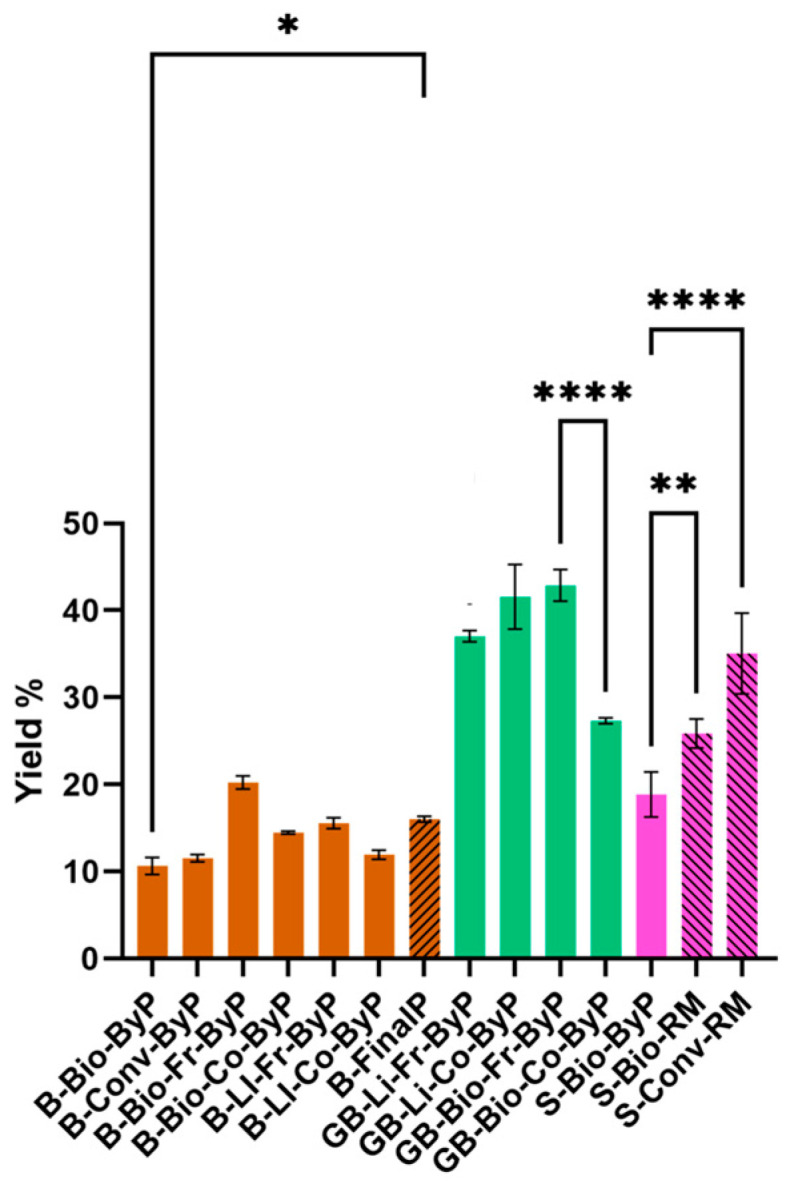
Yield (%) of extracts obtained via UAE from legume by-products (ByP) and final products (FinalP). Results represent the mean ± SD (n = 3). **** *p* < 0.0001, ** *p* < 0.01, * *p* < 0.05.

Figure 1 shows a comparison of extract yield values (%) obtained from bean by-products (brown full bars) and the respective final product (brown striped bar). For soy, the graph compares the extract yield values (%) between the soy by-product (pink full bar) obtained after pressing during the industrial process and the seeds (RM in pink striped bars). In the case of green beans, a comparison is reported between the different by-products analyzed in the project (all green full bars). Regarding bean samples, the results demonstrate that the amount of extracts obtained from the by-products is only slightly lower than that of the corresponding final product. Moreover, the fresh biological by-product (B-Bio-Fr-ByP) even shows a higher yield value (%). This suggests that the extractable portion from the by-product and the final product is similar. As for the green beans by-products, no significant differences are observed except for the cooked biological by-product (GB-Bio-Co-ByP), which has the lowest yield values (%) among green beans. This difference could be attributed to the composition of this sample. Lastly, soy seeds (S-Bio-RM and S-Conv-RM) were found to have higher yield values (%) than the by-product. In this case, both the industrial procedure for obtaining soy milk and the composition of the sample itself may have affected the % yield of dry extract obtained.

### 3.2. Characterization of Legume UAE Extracts by UHPLC-DAD-ESI-MS^n^

Many methods are already employed for the separation and identification of bioactive compounds, such as polyphenols, including Ultra-High Performance Liquid Chromatography (UHPLC), Gas Chromatography-Mass Spectrometry (GC-MS) and Attenuated Total Reflection Infrared Spectroscopy (ATR-FTIR) [9]. In our study, we optimized and applied the UHPLC-DAD-ESI-MS^n^ method. The combination of Ultra-High-Performance-Liquid-Chromatography and an MS detector allowed rapid chromatographic runs with minimal solvent and energy consumption while maintaining high resolution. Furthermore, the MS detector enabled the identification of many polyphenolic compounds present in all UAE bean, soy and green bean extracts.

**Figure 2:**
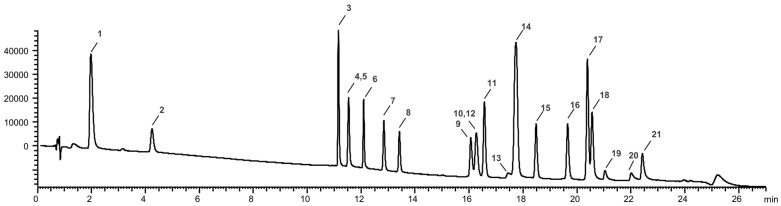
Chromatographic separation (λ = 280 nm) of the 21-standard polyphenol mixture.

A reversed-phase gradient elution was optimized to separate and identify 21 standard polyphenols (Figure 2, Table S2). The standard mixture used was composed of gallic acid (1), protocatechuic acid (2), catechin (3), caffeic acid (4), chlorogenic acid (5), epicatechin (6), p-coumaric acid (7), ferulic acid (8), isoquercitrin (9), rutin trihydrate (10), hyperoside (11), phloridzin dihydrate (12), myricetin (13), quercitrin (14), daidzein (15), naringenin (16), genistein (17), hesperetin (18), naringenin chalcone (19), kaempferol (20) and apigenin (21).

This LC-MS chromatographic method was applied to determine the qualitative profile and the relative amount of polyphenols in the legume UAE extracts under investigation (green beans, beans and soy).

Qualitative analysis of polyphenols in legume UAE extracts was performed using a combination of retention time, UV-Vis spectra, and MS fragmentation patterns. The MS analysis, in both positive and negative ion modes, facilitated the identification of compounds by comparing their MS spectra to those of standards and the literature data.

A semi-quantitative analysis (see Section 2.6) was then performed to estimate the relative amount of individual polyphenols. This analysis enabled a comparative assessment of different sample types, including fresh and cooked products, as well as samples from various cultivation methods (biological, LI, and conventional).

#### 3.2.1. Green Bean Samples (*Phaseolus vulgaris* L.)

The green bean samples include fresh and cooked by-products taken before and after cooking during the industrial production of frozen legumes. As the varieties grown are the same in both LI and biological systems, with the difference that the seeds are not treated at all in the latter case, we can affirm that both cooked and fresh by-products have been subjected to biological and LI cultivation.

The pattern of mass spectra was analyzed in negative mode [M-H]^−^. The results of the UHPLC-DAD-ESI-MS^n^ qualitative analysis (see Table S3 in the Supplementary Materials) demonstrate that green beans are rich in flavonols, a finding that is consistent with previous studies found in the literature. Indeed, as reported by Abu-Reidah et al. [18], flavonoids and their derivatives were identified as the most prominent compounds in all green bean UAE extracts analyzed, underscoring the significance of this class of phenolic compounds in the phytochemical profile of this legume.

**Figure 3:**
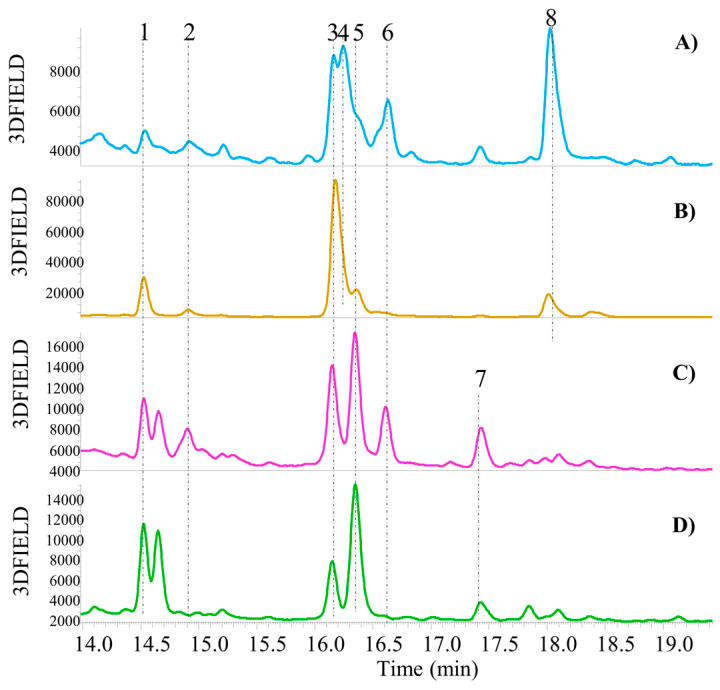
Qualitative UHPLC-DAD analysis (chromatograms recorded at λ = 330 nm) of green bean by-products (method described in Section 2.5). Zoomed-in view of the chromatogram between 13.98 and 19.42 min; (**A**) GB-Fr-LI-ByP; (**B**) GB-Co-LI-ByP; (**C**) GB-Fr-Bio-ByP; (**D**) GB-Co-Bio-ByP; 1 = Quercetin 3-O-xylosylrutinoside; 2 = Quercetin 3-O-vicianoside; 3 = Kaempferol 3-O-xylosylrutinoside; 4 = Quercetin 3-O-glucuronide; 5 = Quercetin 3-O-rutinoside (Rutin); 6 = Kaempferol 3-O-sambubioside; 7 = Kaempferol 3-O-rutinoside; 8 = Kaempferol 3-O-glucuronide.

From a qualitative perspective, no notable differences were observed between the fresh and cooked by-products derived from biological cultivation (Figure 3A,B) and fresh and cooked by-products derived from LI cultivation (Figure 3C,D).

However, a notable distinction was observed between green bean by-products derived from biological cultivation (GB-Co-Bio-ByP and GB-Fr-Bio-ByP) and green bean by-products from LI cultivation (GB-Co-LI-ByP and GB-Fr-LI-ByP). Indeed, both fresh and cooked LI by-products (A-B samples of Figure 3) are devoid of the presence of a compound whose precursor ion is *m*/*z* 593, which would appear to be kaempferol 3-O-rutinoside (peak 7, Figure 3), as previously reported in the literature [18].

In contrast, both biological by-products (C-D samples of Figure 3) are devoid of the presence of peak 4 (see Figure 3), which is identified as quercetin 3-O-glucuronide, as it has a parent ion of *m*/*z* 477 [18], and peak 8 (see Figure 3), which is identified as kaempferol 3-O-glucuronide, as it has a parent ion of *m*/*z* 461.

However, regarding all other compounds, the MS spectra have revealed the presence of the parent ion at *m*/*z* 741 (peak 1, Figure 3), which is identified as quercetin 3-O-xylosylrutinoside. Peak 2 (see Figure 3) at *m*/*z* 595 has been demonstrated to be quercetin 3-O-vicianoside, which is present even in the biological fresh by-product but absent in the cooked one. The compound with *m*/*z* 725 (peak 3, Figure 3) has been identified as kaempferol 3-O-xylosylrutinoside, as previously described [18]. The identification of quercetin 3-O-rutinoside (rutin), corresponding to peak 5 (see Figure 3), was confirmed by comparison with the reference molecule available in our laboratories. According to the literature [18], this compound was identified in all green bean UAE extracts, with the exception of the LI-cooked by-product. The parent ion of *m*/*z* 579 observed for compound 6 has been proposed to be kaempferol 3-O-sambubioside (see Figure 3).

**Figure 4:**
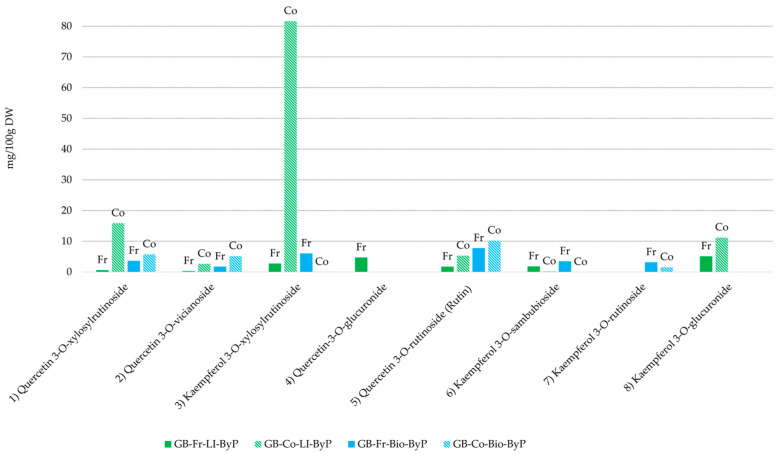
Polyphenol content (semi-quantitative UHPLC-DAD analysis) in green bean by-products from biological (blue) and LI (green) cultivation. Co = Cooked, Fr = Fresh.

A semi-quantitative analysis of the green bean by-products was also carried out, and the results are shown in Figure 4. Since a comprehensive quantitative analysis was not conducted, standard deviations (SDs) cannot be provided for the data presented in the bar graphs in Figure 4. The bar graph illustrates a comparative analysis of the polyphenol content in fresh and cooked green bean by-products from biological and LI cultivation.

A marked increase in specific polyphenols was observed in cooked by-products when compared to fresh by-products. The most striking increase was observed for kaempferol 3-O-xylosylrutinoside (compound 3, Figure 4) in the cooked LI by-product (GB-Co-LI-ByP) compared to the fresh counterpart (GB-Fr-LI-ByP). A similar pattern is observed for compound 1, quercetin-3-O-xylosylrutinoside, and compound 5, rutin (see Figure 4), which are present in higher amounts in both cooked by-products (GB-Co-LI-ByP and GB-Co-Bio-ByP) compared to their fresh counterparts (GB-Fr-LI-ByP and GB-Fr-Bio-ByP). The elevated concentration of polyphenols in cooked green bean by-products relative to their fresh counterparts can be ascribed to the structural alterations induced by cooking, which are likely to disrupt cell wall integrity and facilitate the diffusion of these compounds. These observations are consistent with the findings of previous research, which have demonstrated that cooking methods can influence the nutritional composition of vegetables, including green beans, resulting in alterations to the level of specific compounds such as polyphenols [19].

On the contrary, some compounds are no longer present in the cooked by-products, including quercetin-3-O-glucuronide (peak 4, Figure 4) and kaempferol 3-O-sambubioside (peak 6, Figure 4) in the GB-Co-Bio-ByP UAE extract. This outcome is to be expected, given that the cooking process is likely to result in the degradation or hydrolysis of the sugar chains of these two compounds, in direct opposition to what we observed before. Indeed, previous research has demonstrated that various processing conditions can significantly impact the degradation of phenolic compounds in common beans, with increased soaking time and water temperature accelerating polyphenol degradation [20].

Thus, there are several mechanisms involved in thermal processes that can influence the increase or decrease of certain compounds, such as polyphenols, in vegetables.

A significant finding from our semi-quantitative analysis of green bean by-products was the presence of some polyphenols in all UAE extracts studied, particularly quercetin-3-O-rutinoside, which has been demonstrated to possess cardioprotective effects [21]. Additionally, the presence of quercetin glucuronide, known for its anti-vascular properties [22], further highlights the potential health benefits of these by-products.

#### 3.2.2. Bean Samples (*Phaseolus vulgaris* L.)

In order to evaluate the potential of bean by-products, qualitative and semi-quantitative analyses were conducted to compare bean by-products derived from different production stages with the final product.

In the case of bean by-products, the most rudimentary waste material obtained from the initial stages of the industrial process, defined as B-Bio-ByP and B-Conv-ByP, underwent UHPLC-DAD-MS^n^ analysis. This material is characterized by the presence of a multitude of foreign bodies, including branches and leaves. Furthermore, by-products from collection points close to the final product were also analyzed, which therefore appear to be less contaminated by foreign bodies than those previously mentioned. Among them, there are the biological by-products (B-Fr-Bio-ByP and B-Co-Bio-ByP) and the LI by-products (B-Fr-LI-ByP and B-Co-LI-ByP), while the final product (B-FinalP) corresponds to frozen beans.

**Figure 5:**
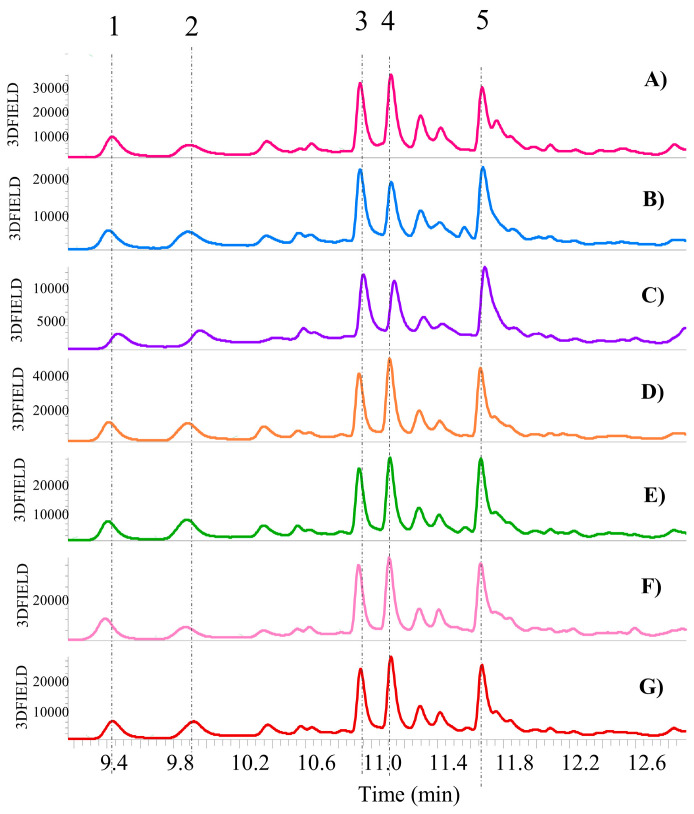
Qualitative UHPLC-DAD analysis (chromatograms recorded at λ = 330 nm) of bean by-products (method described in Section 2.5). Zoomed-in view of the chromatogram between 9.16 and 12.91 min; (**A**) B-FinalP; (**B**) B-Bio-ByP; (**C**) B-Conv-ByP; (**D**) B-Fr-Bio-ByP; (**E**) B-Co-Bio-ByP; (**F**) B-Fr-LI-ByP; (**G**) B-Co-LI-ByP; 1, 2, 3, 4, 5 = Feruloyl glucaric/galactaric acid derivative.

A comparison of the chromatograms of all the bean UAE extracts is shown in Figure 5. From a qualitative standpoint, the phenolic composition of all bean UAE extracts is comparable, including both by-products and the final product. Specifically, the presence of five distinct main compounds, with retention times of 9.47, 9.96, 10.95, 11.14, and 14.69 min (see Table S4), was identified in all bean UAE extracts. The peaks exhibit the same absorption spectra (λ = 326 nm) and identical mass spectra patterns (see Table S4 in Supplementary Materials), with a parent ion of *m*/*z* 385 in negative mode [M-H]^−^. It has been postulated that these compounds are derivatives of ferulic acid coupled with glucaric or galactaric acid, as previously described by Nguyen et al. [23].

The qualitative analysis revealed a promising polyphenol profile in bean by-products, notably the presence of feruloylglucaric acid derivatives. As demonstrated by Walaszek et al. [24], these compounds have shown significant potential in lowering serum cholesterol levels in rats. Thus, bean by-products could serve as a valuable source of natural anti-cholesterolemic agents, transforming waste into a resource with significant health benefits.

**Figure 6:**
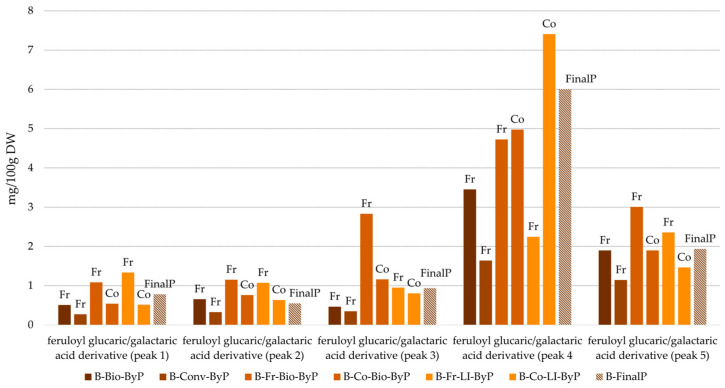
Polyphenol content (semi-quantitative UHPLC-DAD analysis) in bean samples from biological, conventional and LI cultivation. Co = Cooked, Fr = Fresh. By-product = full bars; Final product = striped bars.

As previously mentioned, five compounds, all derivatives of ferulic acid coupled with galactaric or glucaric acid, were identified across all bean UAE extracts. Figure 6 presents a bar graph illustrating the relative amount of these compounds in the analyzed UAE extracts. Since a comprehensive quantitative analysis was not conducted, standard deviations (SDs) cannot be provided for the data presented in the bar graphs in Figure 6. Despite the qualitative similarity in polyphenol composition, the semi-quantitative analysis highlighted significant variations in the amount of these compounds across the different bean by-products.

A comparative analysis was conducted between fresh and cooked bean by-products, and unlike green bean by-products, no significant differences were observed between the two forms. In fact, fresh by-products consistently exhibited higher concentrations of polyphenols than their cooked counterparts (as is the case for peaks 1, 2, 3 and 5, Figure 6). This finding suggests that the cooking process may lead to the extraction of certain polar compounds into the cooking water. This result is in line with previous studies on common beans, which demonstrated that various processing conditions, particularly extended soaking times and high water temperatures, can significantly accelerate polyphenol degradation [20].

Conversely, peak 4 (see Figure 6) exhibits a different trend, with cooked bean by-products displaying slightly higher concentrations than their fresh counterparts. This finding aligns with previous research demonstrating that specific cooking conditions can lead to an increase in certain polyphenol compounds within plant matrices [19]. In conclusion, similar to green bean by-products, the thermal processing of bean by-products significantly impacts their polyphenol profile, leading to both increases and decreases in specific compounds. A detailed quantitative analysis will be performed in future studies to further elucidate these findings.

Furthermore, the study undertook a comparative analysis of the three distinct cultivation methods: biological, conventional, and LI. Figure 6 reveals that peak number 4 (which is one of the feruloylglucaric acid derivatives) is consistently the most abundant across all analyzed by-products. This finding suggests that cultivation practices may influence the accumulation of specific polyphenols. Previous research, such as that conducted by Monika Kalinowska et al. [25], supports this hypothesis, demonstrating that different cultivation methods can significantly impact polyphenol content. It is plausible that reduced pesticide use in biological, LI or sustainable cultivation could contribute to variations in polyphenol profiles. A closer examination of Figure 6 reveals a notable trend: LI and biological by-products exhibit higher polyphenol amounts compared to conventional by-products, highlighting the potential of sustainable agriculture to produce crops with enhanced nutritional value.

A significant finding from this study is the elevated polyphenol content in by-products compared to the final product, as confirmed by Figure 6. This observation underlines the potential of plant-based waste as a valuable source of bioactive compounds, aligning with the primary objective of this research.

A comparative analysis of green beans and mature bean by-products revealed distinct polyphenol profiles. Green beans, representing the “youngest” stage in bean ripening, were characterized by the presence of glycosylated flavonols, as detailed in Table S3. In contrast, mature beans predominantly contained ferulic acid derivatives, as shown in Table S4. According to the literature [16,26], this difference can be attributed to the plant’s developmental stage and its response to environmental stress. During early development, plants often accumulate flavonoids to protect themselves from UV radiation and oxidative stress. As the plant matures and becomes more resilient, it may shift its metabolic focus toward other defense mechanisms.

Indeed, the existing literature confirms that heat affects the degradation of flavonols [27]. For instance, it has been demonstrated that the application of heat can result in the degradation of rutin, leading to the formation of a mixture comprising protocatechuic acid and other degradation derivatives with reduced antioxidant activity [27,28]. In light of these results, it can be hypothesized that in green beans, which represent the earliest and therefore most vulnerable stage of ripening, polyphenols with higher antioxidant activity, such as flavonols, are present. Conversely, beans exhibit a greater presence of polyphenols with lower antioxidant activity, such as phenolic acids. Once they have reached maturity, beans have a reduced need to actively defend themselves and therefore produce fewer substances. Additionally, it is essential to consider that the mature plant will have endured adverse weather conditions, such as drought or extreme temperatures, which may have caused a degradation of the polyphenol structure, leading to the formation of phenolic acids.

#### 3.2.3. Soy Samples

**Figure 7:**
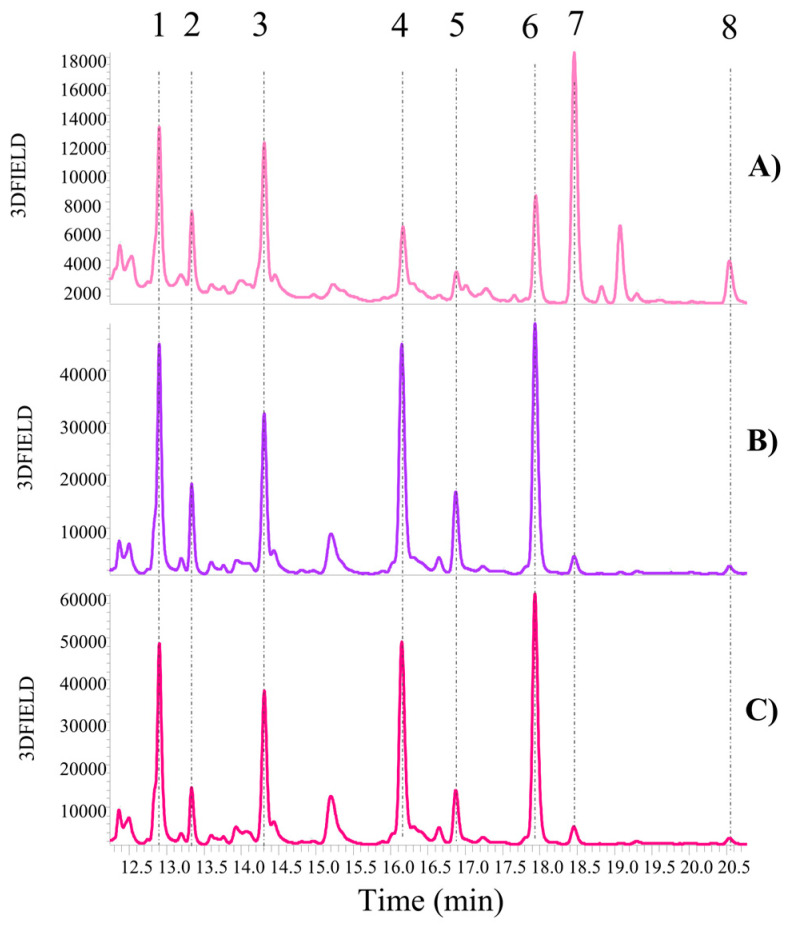
Qualitative UHPLC-DAD analysis (chromatograms recorded at λ = 330 nm) of soy samples (method described in Section 2.5). Zoomed-in view of the chromatogram between 12.24 and 20.77 min; (**A**) S-Bio-ByP; (**B**) S-Bio-RM; (**C**) S-Conv-RM; 1 = Daidzin; 2 = Glycitin; 3 = Genistin; 4 = Malonyl daidzin; 5 = Malonyl glycitin; 6 = Malonyl genistin; 7 = Daidzein; 8 = Genistein.

In the case of soy samples, the qualitative LC-MS analysis (see Figure 7, Table S5) was performed on raw materials, which are seeds, including both biological (S-Bio-RM) and conventional (S-Conv-RM) seeds, as well as biological okara, which is the soy by-product (S-Bio-ByP). The pattern of mass spectra was analyzed in positive mode [M-H]^+^.

According to the literature [29], soy samples are well-established sources of isoflavones and their derivatives. A key distinction of our research is the comparative analysis of soy by-product (okara) and its corresponding raw materials (seeds). Among the by-products investigated in this research, okara stands out as a particularly promising candidate due to its relative purity. The closed-loop nature of the soy milk production process, which involves minimal exposure to external contaminants like stones, branches, or insects, contributes to the cleanliness of the resulting okara.

The qualitative analysis of soy, comprising both seed and waste samples, revealed a comparable isoflavone composition. Among the isoflavones identified, all soy UAE extracts showed the presence of daidzin *m*/*z* 417 (peak 1, Figure 7), glycitin *m*/*z* 447 (peak 2, Figure 7), genistin *m*/*z* 433 (peak 3, Figure 7), malonyl daidzin *m*/*z* 503 (peak 4, Figure 7), malonyl glycitin *m*/*z* 533 (peak 5, Figure 7), and malonyl genistin *m*/*z* 519 (peak 6, Figure 7). The identification of these compounds in our soy UAE extracts was confirmed through a comparison with previously published LC-MS data by Lee et al. [30]. Meanwhile, the identification of daidzein and genistein, with respective parent ions of *m*/*z* 255 and *m*/*z* 271 (peaks 7 and 8, Figure 7), was confirmed by comparison with the standard molecules available in our laboratories.

The qualitative analysis revealed good similarity in the isoflavone profiles of okara (S-Bio-ByP) and soy seeds (S-Conv-RM and S-Bio-RM). This result underlines the potential of okara (S-Bio-ByP) as a valuable source of bioactive compounds, mainly isoflavones. Given the well-documented health benefits of isoflavones, including their role in bone health, okara-derived isoflavones may offer promising therapeutic applications for conditions such as osteoporosis [31].

**Figure 8:**
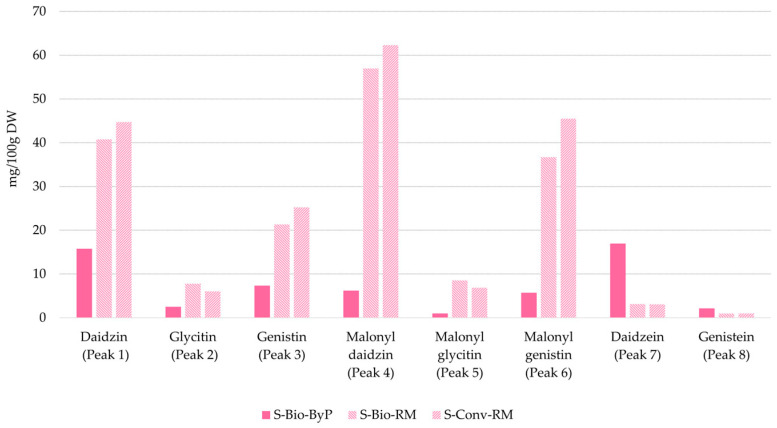
Polyphenol content (semi-quantitative UHPLC-DAD analysis) in soy samples. By-product (okara) = full bars; raw material (seeds) = stripped bars.

Figure 8 shows a comparative analysis of the semi-quantitative data for soy UAE extracts. Since a comprehensive quantitative analysis was not conducted, standard deviations (SDs) cannot be provided for the data presented in the bar graphs in Figure 8. The qualitative analysis demonstrated a comparable polyphenol profile across the seeds (S-Conv-RM and S-Bio-RM) and okara (S-Bio-ByP), whereas the semi-quantitative analysis revealed notable discrepancies in the quantity of specific isoflavones.

A comparative analysis of the amount of individual isoflavones revealed a distinct pattern: glucoside forms (peaks 1–6, Figure 8) were more abundant in seeds (S-Conv-RM and S-Bio-RM), while aglycone forms, such as daidzein (peak 7, Figure 8) and genistein (peak 8, Figure 8), were more concentrated in okara (S-Bio-ByP). This observation is consistent with the existing literature, which has established that soy primarily contains isoflavone glycosides, such as malonyl glucosides and β-glucosides [32]. The increased aglycone content in the by-product can be attributed to thermal processing, which can facilitate the hydrolysis of glycosides into aglycones. Additionally, the extraction of glycosides into soy milk during processing may further contribute to the enrichment of aglycones in the by-product.

Thus, the qualitative and semi-quantitative analyses demonstrated the significant polyphenol content of okara (S-Bio-ByP), including a comparable isoflavone profile to soy seeds. These results highlight the potential of okara as a valuable source of bioactive compounds, transforming a previously unused by-product into a resource with significant health benefits.

### 3.3. Total Phenolic Content (TPC)

**Figure 9:**
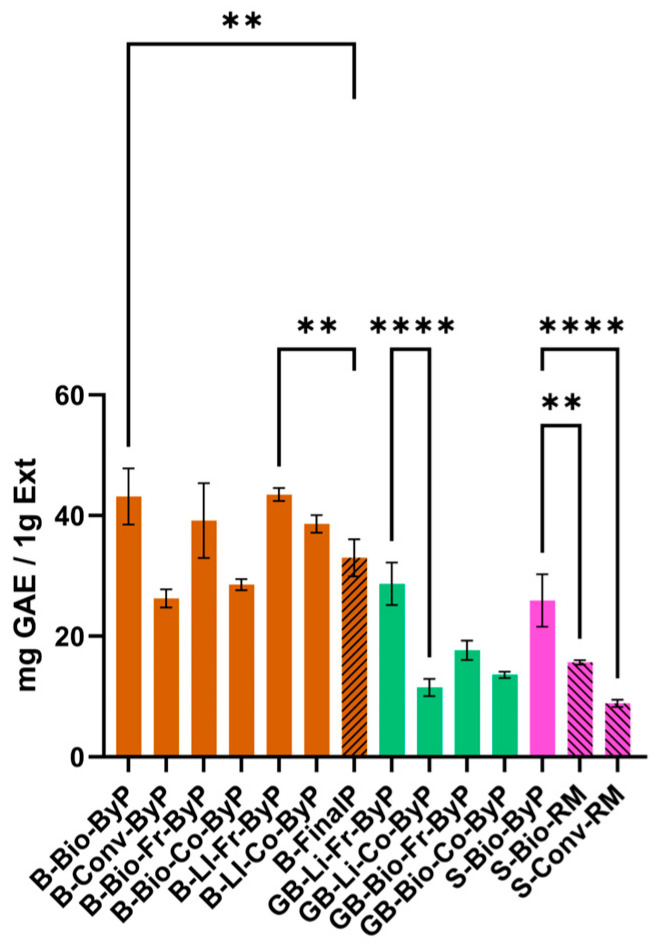
TPC (mg GAE/1 g Ext) of ByP (full bars) and FinalP (stripped bars) extracts obtained via UAE. Data represent mean ± SD (n = 3, each analyzed in duplicate). **** *p* < 0.0001, ** *p* < 0.01.

This study investigated the TPC of by-products from various legumes (beans, green beans, and soy) and compared it to the final product for trade (see Figure 9, Table S1). Our findings revealed considerable variation in polyphenolic content among legume UAE extracts. Interestingly, the TPC found for bean by-products, ranging from 26.27 ± 1.49 mg GAE/1 g Ext to 43.49 ± 1.07 mg GAE/1 g Ext, generally equaled or exceeded that of the final product (33.01 ± 3.08 mg GAE/1 g Ext). This suggests significant retention of polyphenols within the by-products after processing. These results are consistent with previous studies on black bean by-products employing conventional solid-liquid extraction methods, albeit with different solvents [33]. For example, Moreno-García et al. (2022) [33] reported a TPC of 38.41 and 38.60 mg GAE/1 g Ext for black bean by-products, supporting the trends observed in our bean by-product extracts.

Further analysis revealed a slight decrease in the TPC of cooked by-products compared to their fresh counterparts. This reduction is likely attributable to thermal processing during cooking, which can degrade polyphenols [34]. Similar observations have been reported in the literature, with Duan et al. [34] demonstrating a decrease in the TPC of faba leaves and seeds after wet heat treatment. Notably, moist heat treatments, such as boiling, have been shown to significantly reduce TPC across various food types [34]. This observation was further supported by the decrease in TPC of biological green bean by-products after cooking, from 17.68 ± 1.61 to 13.60 ± 0.53 mg GAE/1 g Ext, and LI green bean by-products, from 28.68 ± 3.52 to 11.51 ± 1.43 mg GAE/1 g Ext.

In contrast, okara (S-Bio-ByP) exhibited a significantly higher TPC, 309.6 ± 0.13 mg GAE/100 g DW, compared to the value reported by Vital et al. (2018) [35], which is 130.50 ± 10.20 mg GAE/100 g DW (result was expressed as mg GAE/100 g dry weight (DW) to facilitate comparison with the literature data) obtained by conventional extraction with 50% acetone mixed for 30 min [35]. This discrepancy may be attributed to the use of an ultrasonic bath for the extraction in our study. Compared to conventional solid-liquid mixing, ultrasonic extraction facilitates cell wall rupture and disintegration of the solid matrix, thereby increasing the contact surface between solvent and target compounds and potentially enhancing polyphenol yield.

Concerning different cultivation methods, the data reported in Figure 9 show that for the three types of samples analyzed—green beans, beans, and soy—biological or LI methods generally result in higher amounts of polyphenols compared to conventional ones. This could be explained by the fact that reduced pesticide use might induce polyphenol production as part of the plants’ defense mechanisms.

### 3.4. Total Antioxidant Status (TAS)—ABTS^•+^ Radical Cation Scavenging Activity

The antioxidant activity of the bean by-products, ranging from 162.50 ± 2.75 to 336.33 ± 3.06 µmol TE/g Ext), was comparable to that of the final product (334.33 ± 0.76 µmol TE/g Ext). Notably, B-Bio-ByP exhibited slightly higher antioxidant activity than the final product (B-FinalP), 336.33 ± 3.06 µmol TE/g Ext vs. 334.33 ± 0.76 µmol TE/g Ext respectively. These findings align with previous research. For instance, Moreno-García et al. [33] reported antioxidant activity values of 345.48 and 360.81 µmol TE/g Ext for black bean by-products extracted with a conventional method using 80% methanol and 70% ethanol, respectively. Our results further confirm the potential of bean by-products as a valuable source of antioxidants.

**Figure 10:**
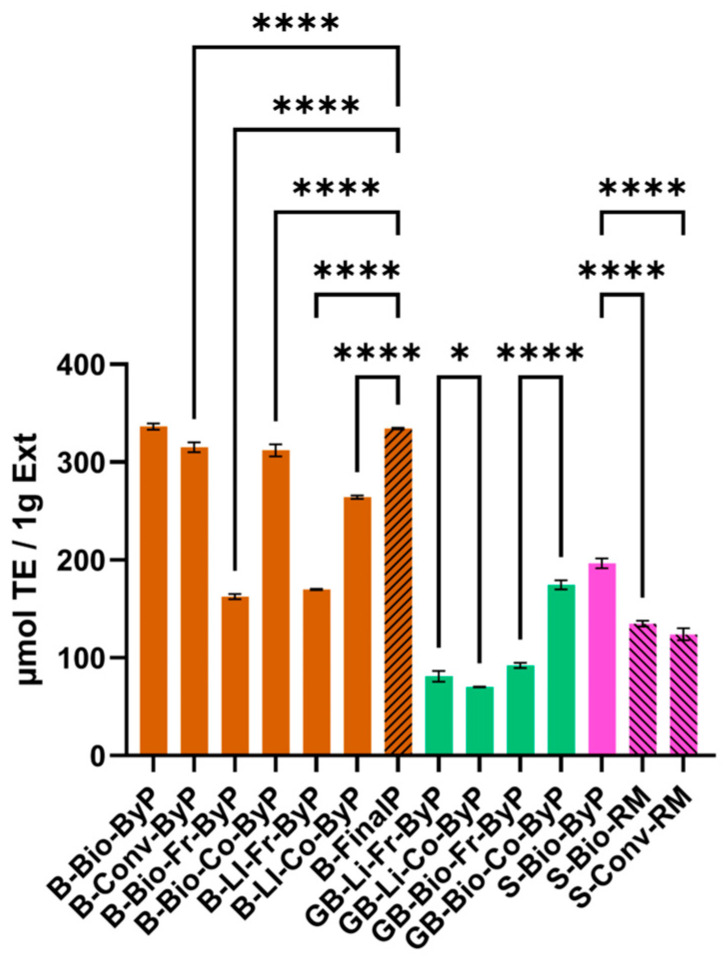
TAS (µmol TE/1 g Ext) of ByP (full bars) and FinalP (stripped bars) extracts obtained via UAE. Data represent mean ± SD (n = 3, each analyzed in duplicate). **** *p* < 0.0001, * *p* < 0.05.

A comparative analysis of fresh and cooked bean by-products revealed a surprising trend: the antioxidant activity was consistently higher in the cooked UAE extracts, regardless of whether they were derived from biological (B-Co-Bio-ByP: 312.00 ± 6.08 µmol TE/g Ext vs. B-Fr-Bio-ByP: 162.50 ± 2.75 µmol TE/g Ext) or LI (B-Co-LI-ByP: 264.25 ± 1.68 µmol TE/g Ext vs. B-Fr-LI-ByP: 169.83 ± 0.63 µmol TE/g Ext) cultivation methods, as reported in Figure 10. Several factors may contribute to this unexpected outcome. Firstly, cooking processes, such as stewing, can inactivate certain substances that inhibit the absorption of antioxidants in fresh beans, thereby enhancing their bioavailability. Secondly, thermal treatment can disrupt the cell wall structure, facilitating the release of antioxidants into the medium. However, it is crucial to acknowledge that the impact of thermal processing on antioxidant activity can vary significantly depending on factors such as processing time, temperature, and the specific compounds present in the food matrix.

For instance, Ng et al. [36] have demonstrated that boiling, in some cases, can significantly enhance the antioxidant activity of certain vegetables, such as broccoli, bitter gourd, and water convolvulus. This highlights the importance of considering the specific vegetable type and cooking method to optimize the preservation or enhancement of its nutritional value.

These findings are further supported by the observed antioxidant activity in green bean by-products (see Figure 10). Consistent with previous observations, cooked green beans exhibited higher antioxidant activity compared to their fresh counterparts. This suggests that, in the specific case of green beans, steaming may enhance their antioxidant potential.

In the case of soy, to validate our findings, a comparative analysis of the antioxidant activity of S-Bio-ByP, a by-product of soy milk production, was conducted with previously published data. The antioxidant activity was assessed using the ABTS radical scavenging assay, and the results were expressed as inhibition percentage (I%) to facilitate comparison with the literature data. Our study yielded an I% of 24.54, which is slightly lower than the value of 37.84 ± 3.14 reported by Vital et al. [35]. However, this difference is considered minor and does not significantly deviate from the general trend reported in the literature.

Interestingly, our results indicate that okara (S-Bio-ByP) exhibits higher antioxidant activity than the original soy seeds (196.61 ± 5.01 µmol TE/g Ext vs. 134.86 ± 2.93 and 123.97 ± 6.16 µmol TE/g Ext respectively). This unexpected result can be attributed to the concentration of bioactive compounds that occurs during the processing of soy milk. Polyphenols, known for their potent antioxidant properties, tend to accumulate in the solid fraction (okara) during the extraction process. This concentration effect likely contributes to the enhanced antioxidant activity observed in okara.

To further support this hypothesis, it is possible to observe that TPC results revealed a lower concentration of polyphenols in soy seeds, S-Bio-RM and S-Conv-RM, (15.66 ± 0.37 mg GAE/1 g Ext and 8.89 ± 6.03 mg GAE/1 g Ext), compared to okara (S-Bio-ByP) (25.94 ± 4.35 mg GAE/1 g Ext), in line with the observed higher antioxidant activity in the latter.

### 3.5. Protein Content by the Kjeldahl Method

**Figure 11:**
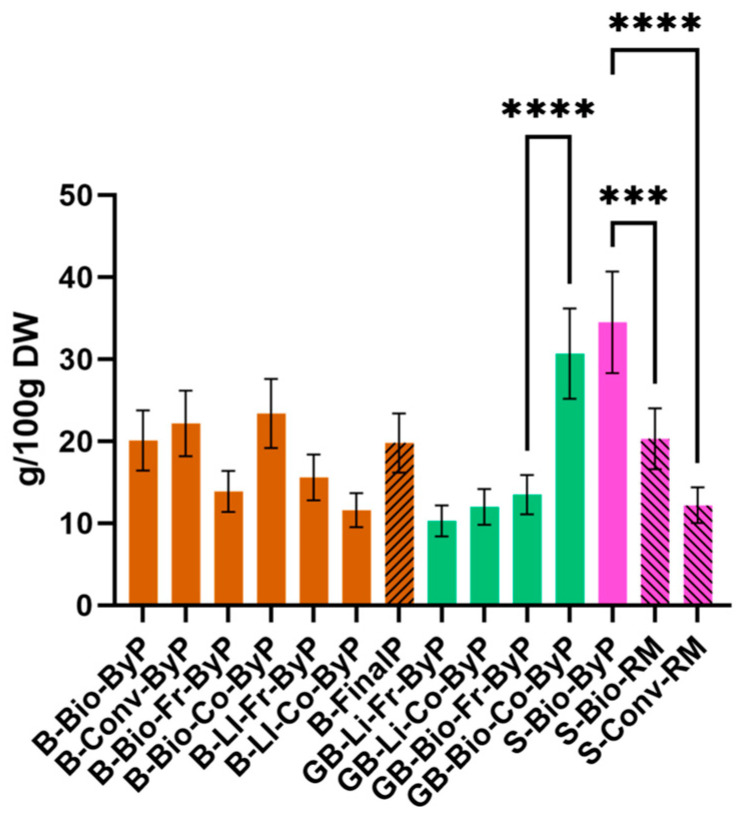
Protein content (g/100 g DW) of ByP (full bars) and FinalP (stripped bars) determined by the Kjeldahl method. Data represent mean ± SD (n = 2). **** *p* < 0.0001, *** *p* < 0.005.

The protein content of each sample was determined using the Kjeldahl method, and the results expressed as g of protein in 100 g of dry weight (DW) are presented in Figure 11.

All bean by-products revealed a wide range, going from 11.52 ± 0.22 g/100 g DW to 23.40 ± 4.20 g/100 g DW of protein. Notably, some bean by-products exhibited protein content lower than, equal to, or even exceeding that of the corresponding final product (19.80 ± 3.60 g/100 g DW of protein). To validate these findings, a literature review was conducted. A study by Mariscal-Moreno et al. [37] reported a protein content of 23.94% in black bean (*Phaseolus vulgaris* L.) flour. This value aligns well with the higher end of the observed protein content range, further supporting the potential of bean by-products as valuable protein sources.

Concerning green bean by-products, the obtained results revealed a protein content ranging from 10.30 ± 1.90 g/100 g DW to 30.70 ± 5.50 g/100 g DW, comparable to that of bean by-products (11.60 ± 2.10 g/100 g DW to 23.40 ± 4.20 g/100 g DW of protein). Especially, the cooked biological by-product (GB-Co-Bio-ByP) exhibited the highest protein content among all bean and green bean samples, including the bean final product, reaching 30.70 ± 5.50 g/100 g DW. The current results validate those of previous research. For instance, published studies [38] have reported protein content in green bean by-product of 22 g/100 g DW, exceeding that of beans (*Phaseolus vulgaris* L.) of 20.9 g/100 g DW. This comparison with the established literature further confirms the protein-rich nature of the examined green bean by-products.

Among all investigated legume by-products, okara (soy by-product) demonstrated the highest protein content, reaching 34.50 ± 6.20 g/100 g DW. This is supported by previous research. Asghar et al. [39] reported a protein content of 34.15% in dry okara, further validating our result. These results underscore the potential of soy by-products as a valuable source of protein, potentially offering greater nutritional benefits than the seeds themselves.

Concerning the cultivation method, no significant differences were observed between B-ByP and B-Final-P. However, for green beans and soy, bio cultivation resulted in a higher protein content in the cooked GB-Bio-Co-ByP and S-Bio-RM samples.

The legume by-products studied, especially okara (S-Bio-ByP), could be widely used in the cosmetics industry due to their high protein content. Indeed, proteins contribute to skin hydration by increasing water content in the stratum corneum, reducing dehydration, and improving barrier function. They also hydrate the scalp, making them suitable for hair care products [40,41].

## 4. Conclusions

This research project innovatively investigates the impact of cultivation type, thermal processing, and by-product origin (waste or marketable product) on the phenolic profiles and protein content of agri-food by-products. All the analyses carried out in this study confirmed the rich content of bioactive molecules in legume by-products, specifically those analyzed.

The advanced UHPLC-DAD-ESI-MS^n^ chromatographic method proved to be a necessary procedure to carry out the qualitative and semi-quantitative analyses of polyphenols in legume by-products.

Starting from bean UAE extracts, LC-MS qualitative analysis revealed a similar qualitative phenolic profile across all samples, with feruloylglucaric acid derivatives as the major compounds. Surprisingly, by-products generally exhibited higher polyphenol content than the final product, suggesting their potential as a valuable source of polyphenolic compounds. Regarding cultivation, sustainable methods (biological and LI) were associated with higher polyphenol levels in bean UAE extracts compared to conventional methods. Thermal processing slightly reduced polyphenol content in cooked by-products compared to their fresh counterparts, except for a specific compound, which is peak 4 in Figure 6, which is unexpectedly more concentrated in the cooked by-product. This trend was further confirmed by TPC results, which were comparable between by-products and the final product (ranging from 26.27 ± 1.49 to 43.49 ± 1.07 mg GAE/1 g Ext for by-product; 33.01 ± 3.08 mg GAE/1 g Ext for the final product), with a slight decrease in cooked bean by-products. However, Total Antioxidant Activity (TAS) results opposed TPC findings, indicating a significant increase in antioxidant activity in cooked by-products, regardless of cultivation method.

Regarding green bean by-products, the LC-MS qualitative analysis revealed that they are rich in flavonols with a comparable profile between all UAE extracts. While a notable qualitative distinction was observed between the type of cultivation: biological green bean by-products (GB-Co-Bio-ByP and GB-Fr-Bio-ByP) are devoid of the presence of quercetin 3-O-glucuronide and kaempferol 3-O-glucuronide; LI green bean by-products (GB-Co-LI-ByP and GB-Fr-LI-ByP) are devoid of the presence of kaempferol 3-O-rutinoside. Focusing on the polyphenolic content, thermal processing significantly enhanced the polyphenol content of by-products, as evidenced by the increased levels of specific compounds such as kaempferol 3-O-xylosylrutinoside in cooked samples. While Total Antioxidant Activity (TAS) results further support this trend, demonstrating an increased antioxidant activity in cooked biological by-products (GB-Co-Bio-ByP, 174.47 ± 4.74 µmol TE 1 g^−1^ Ext), TPC analysis revealed a decrease in total phenolic content for both biological and LI green bean by-products following cooking.

The LC-MS qualitative analysis mentioned above revealed an interesting difference in the phenolic profiles between green beans (young stage) and beans (mature stage). While green beans predominantly contained glycosylated flavonoids, mature beans were characterized by feruloylglucaric acid derivatives. This difference likely reflects the plant’s developmental stage and its response to environmental stress. Younger plants, such as green beans, often accumulate flavonoids as a defense mechanism against UV radiation and oxidative stress. As plants mature and become more resilient, they may shift their metabolic focus to other defense strategies.

Concerning soy UAE extracts, LC-MS qualitative analysis showed a comparable richness of isoflavones in both soy seeds (S-Bio-RM and S-Conv-RM) and okara (S-Bio-ByP). The LC-MS semi-quantitative analysis revealed a different distribution of isoflavones in the soy UAE extracts: seeds (S-Bio-RM and S-Conv-RM) were richer in glucosidic forms of isoflavones, whereas okara (S-Bio-ByP) showed higher concentrations of aglycone forms (such as daidzein and genistein). Surprisingly, okara (S-Bio-ByP) exhibited higher TAS (196.61 ± 5.01 µmol TE 1 g^−1^ Ext) and TPC values (25.94 ± 4.35 mg GAE/1 g Ext) than the original soy seeds (S-Bio-RM and S-Conv-RM), underlining its potential as a valuable source of antioxidants.

Finally, the protein content, determined by applying the Kjeldahl method, varied significantly across the legume by-products studied. Some by-products, B-Conv-ByP (22.20 ± 4.00 g/100 g DW), even exhibited higher protein content than the final product, B-FinalP (19.80 ± 3.60 g/100 g DW). Concerning green bean by-products, notably, the cooked biological green bean by-product (GB-Co-Bio-ByP) exhibited the highest protein content (30.70 ± 5.50 g/100 g DW) among all bean and green bean samples, including the bean final product. Of all the legume by-products analyzed, okara (S-Bio-ByP) exhibited the highest protein content, reaching 34.50 ± 6.20 g/100 g DW. These findings underscore the potential of soy by-products (S-Bio-ByP) as a valuable protein source, potentially offering greater nutritional benefits than the seeds themselves.

To conclude, all the results suggest that the UAE legume by-product extracts (beans, green beans and soy) could be used as active ingredients in nutraceutical and dietary supplement formulations thanks to their content of polyphenols and proteins. These UAE extracts from legume waste could contribute to the prevention or treatment of certain diseases in nutraceutical products and address micronutrient or macronutrient deficiencies in food supplements. Therefore, the incorporation of polyphenol extracts for their antioxidant properties and protein extracts for their nutritional value into nutraceutical formulations holds significant promise in line with the circular economy perspective.

## Figures and Tables

**Table 1:** A comprehensive list of agri-food samples: categories, type of cultivation and relative acronym.

Sample		Acronym
Beans	Biological by-product	B-Bio-ByP
	Conventional by-product	B-Conv-ByP
	Final product	B-FinalP
	Biological fresh by-product	B-Fr-Bio-ByP
	Biological cooked by-product	B-Co-Bio-ByP
	LI fresh by-product	B-Fr-LI-ByP
	LI cooked by-product	B-Co-LI-ByP
Green Beans	LI fresh by-product	GB-Fr-LI-ByP
	LI cooked by-product	GB-Co-LI-ByP
	Biological fresh by-product	GB-Fr-Bio-ByP
	Biological cooked by-product	GB-Co-Bio-ByP
Soy	Biological raw material	S-Bio-RM
	Conventional raw material	S-Conv-RM
	Biological by-product	S-Bio-ByP

## Data Availability

All data is contained within the article.

## Supplementary Materials

The following supporting information can be downloaded at: https://www.mdpi.com/article/10.3390/antiox13121531/s1, Table S1. Gravimetric Determination, Total Phenolic Content (TPC), Total Antioxidant Status (TAS) and Kjeldahl method data; Table S2. Retention time (RT), molecular ion [M-H]^−^, MS^n^ fragmentation patterns, molecular weight, and concentration for 21 polyphenols standard analyzed using UHPLC-DAD-ESI-MS^n^; Table S3. Qualitative LC-MS analysis of polyphenols in green beans by-products; Table S4. Qualitative LC-MS analysis of polyphenols in bean products; Table S5. Qualitative LC-MS analysis of polyphenols in soy products.

## Abbreviations

UAE	Ultrasound-assisted extraction
HPLC-DAD	High-Performance Liquid Chromatography–Diode Array Detector
UHPLC-DAD-ESI-MS^n^	Ultra High Performance Liquid Chromatography-Diode Array Detector-Electrospray Ionization-Mass Spectrometry
LC-MS	Liquid Chromatography-Mass Spectrometry
TPC	Total phenolic content
TAS	Total antioxidant status
TE	Trolox equivalent
ByP	By-products
FinalP	Final products
RM	raw material
Ext	dry extract
DW	dry weight
GAE	gallic acid equivalent
SD	standard deviation
LI	lotta Integrata
