# Supplementary material for: Valorization of Legume By-Products Based on Polyphenols and Protein Contents for Potential Nutraceutical Applications
Source: Antioxidants (Basel). 2024 Dec 14;13(12):1531. doi: 10.3390/antiox13121531 (PMC11727440; doi:10.3390/antiox13121531)
Supplement: Supplementary file 1 [file antioxidants-13-01531-s001.zip › antioxidants-3331085-supplementary.pdf]

Table S1. Gravimetric Determination, Total Phenolic Content (TPC), Total Antioxidant Status (TAS) and Kjeldahl method data

	Total Phenolic Content (TPC)	Total Antioxidant Status (TAS)	Gravimetric Determination	Protein Content Kjeldahl method
	Average mg GAE 1g-1 Ext ±SD	Average μmol TE 1g-1 Ext ±SD	Yield (%)	Average g 100g $^{-1}$ DW ±SD
	(n=3)	(n=3)	(n=3)	(n=3)
B-Bio-ByP	43.18±4.66E+00	336.33±3.06E+00	10.64±4.89E-01	20.10±3.60E+00
B-Conv-ByP	26.27±1.49E+00	315.00±5.00E+00	11.52±2.17E-01	22.20±4.00E+00
B-Fr-Bio-ByP	39.17±6.19E+00	162.50±2.75E+00	20.25±7.52E-01	13.90±2.50E+00
B-Co-Bio-ByP	28.55±9.30E-01	312.00±6.08E+00	14.47±1.60E-01	23.40±4.20E+00
B-Fr-LI-ByP	43.49±1.07E+00	169.83±6.34E-01	15.58±6.26E-01	15.60±2.80E+00
B-Co-LI-ByP	38.63±1.46E+00	264.25±1.68E+00	11.92±5.14E-01	11.60±2.10E+00
B-FinalP	33.01±3.08E+00	334.33±7.64E-01	16.01±1.65E-01	19.80±3.60E+00
GB-Fr-LI-ByP	28.68±3.52E+00	81.08±5.45E+00	37.03±6.57E-01	10.30±1.90E+00
GB-Co-LI-ByP	11.51±1.43E+00	70.20±3.87E-01	41.57±3.72E+00	12.00±2.20E+00
GB-Fr-Bio-ByP	17.68±1.61E+00	92.13±2.72E+00	42.87±1.82E+00	13.50±2.40E+00
GB-Co-Bio-ByP	13.60±5.26E-01	174.47±4.74E+00	27.31±3.14E-01	30.70±5.50E+00
S-Bio-ByP	25.94±4.35E+00	196.61±5.01E+00	18.85±1.29E+00	34.50±6.20E+00
S-Bio-RM	15.66±3.68E-01	134.86±2.93E+00	25.85±8.37E-01	20.30±3.70E+00
S-Conv-RM	8.89±6.03E-01	123.97±6.16E+00	35.05±4.64E+00	12.20±2.20E+00

Table S2. Retention time (RT), molecular ion [M-H]-, MS^n fragmentation patterns, molecular weight, and concentration for 21 polyphenols standard analyzed using UHPLC-DAD-ESI-MS^n

Substance	RT [min]	Molecular Ion [m/z]	MS^n [m/z], rel. int. (%)	Molecular Weight [g/mol]	Compound	μg mL-1
1	1.98	169 [M-H]-	MS^2[169]: 125 (100)<br>MS^3[125]: 81 (100), 125 (65), 97 (60)	170	Gallic acid	28.13
2	4.25	153 [M-H]-	MS^2[153]: 109 (100)<br>MS^3[109]: 109 (100), 112 (55)	154	Protocatechuic acid	11.25
3	11.16	289 [M-H]-	MS^2[289]: 245 (100), 205 (35), 179 (15)<br>MS^3[245]: 203 (100), 227 (30), 187 (20)	290	(+)-Catechin	62.50
4	11.51	179 [M-H]-	MS^2[179]: 135 (100)<br>MS^3[135]: 90 (100)	180	Caffeic acid	9.38
5	11.57	353 [M-H]-	MS^2[353]: 191 (100), 179 (48), 173 (30)	354	Chlorogenic acid	3.75

			MS^3[191]: 127 (100), 173 (90), 85 (60), 93 (45), 110 (35)			
6	12.09	289 [M-H]-	MS^2[289]: 245 (100), 205 (35), 179 (15) MS^3[245]: 203 (100), 227 (30), 187 (20)	290	(-)-Epicatechin	28.13
7	12.84	163 [M-H]-	MS^2[163]: 119 (100)	164	p-coumaric acid	3.50
8	13.42	193 [M-H]-	MS^2[193]: 149 (100), 178 (55), 134 (20) MS^3[149]: 134 (100)	194	Ferulic acid	5.63
9	16.08	463 [M-H]-	MS^2[463]: 301 (100) MS^3[301]: 179 (100), 273 (15)	464	Isoquercitrin	18.75
10	16.29	609 [M-H]-	MS^2[609]: 301 (100) MS^3[301]: 179 (100), 273 (15)	610	(+)-Rutin trihydrate	37.50
11	16.57	463 [M-H]-	MS^2[463]: 301 (100) MS^3[301]: 179 (100), 273 (15)	464	Hyperoside	37.50
12	16.79	481 [M+HCOO]- 435 [M-H]-	MS^2[435]: 273 (100)	472	Phloridzin dihydrate	28.13
13	17.47	317 [M-H]-	MS^2[317]: 179 (100), 191 (15) MS^3[179]: 151 (100)	318	Myricetin	19.80
14	17.78	447 [M-H]-	MS^2[447]: 301 (100) MS^3[301]: 179 (100), 151 (65)	448	Quercitrin	56.25
15	18.49	253 [M-H]-	MS^2[253]: 253 (100), 209 (25), 197 (10) MS^3[253]: 253 (100), 209 (30), 181 (25), 169 (10)	254	Daidzein	17.00
16	19.65	271 [M-H]-	MS^2[271]: 151 (100) MS^3[151]: 107 (100)	272	Naringenin	7.50
17	20.39	269 [M-H]-	MS^2[269]: 225 (100), 269 (50), 201 (35), 149 (25) MS^3[225]: 181 (100), 197 (40), 169 (15), 225 (10)	270	Genistein	14.58
18	20.56	301 [M-H]-	MS^2[301]: 286 (100), 242 (45), 257 (35), 125 (20), 199 (10) MS^3[286]: 242 (100), 258 (85), 199 (40), 174 (15), 268 (15)	302	Hesperetin	28.20
19	21.08	271 [M-H]-	MS^2[271]: 151 (100), 176 (20) MS^3[151]: 107 (100)	272	Naringenin chalcone	16.50
20	22.05	285 [M-H]-	MS^2[285]: 285 (100), 243 (55), 151 (40)	286	Kaempferol	12.00
21	22.43	269 [M-H]-	MS^2[269]: 225 (100), 269 (55), 201 (35), 149 (25) MS^3[225]: 181 (100), 197 (40), 169 (15)	270	Apigenin	13.20

Table S3. Qualitative LC-MS analysis of polyphenols in green beans by-products.

Substance	RT [min]	Molecular Ion [m/z]	MS^n [m/z], rel. int. (%)	Molecular Weight [g/mol]	Tentative Identification
1	14.53	741 [M-H]$^-$	MS^2[741]: 253 (100) MS^3[253]: 235 (100)	742	Quercetin 3-O-xylosylrutinoside [18]
2	14.91	595 [M-H]$^-$	MS^2[595]: 300 (100), 445 (40), 463 (25), 475 (15), MS^3[300]: 271 (100), 255 (50)	596	Quercetin 3-O-vicianoside [18]

3	16.16	725 [M-H]$^-$	MS^2[725]: 575 (100), 285 (40), 593 (35)<br>MS^3[575]: 339 (100), 393 (90), 429 (70), 547 (25)	726	Kaempferol 3-O-xylosylrutinoside [18]
4	16.27	477 [M-H]$^-$	MS^2[477]: 301 (100)<br>MS^3[301]: 179 (100), 150 (60)	478	Quercetin 3-O-glucuronide [18]
5	16.38	609 [M-H]$^-$	MS^2[609]: 301 (100)<br>MS^3[301]: 179 (100), 273 (15)	610	Quercetin 3-O-rutinoside
6	16.63	579 [M-H]$^-$	MS^2[579]: 285 (100), 429 (60), 447 (20), 257 (15)<br>MS^3[285]: 257 (100), 151 (50), 267 (45)	580	Kaempferol 3-O-sambubioside [18]
7	17.43	593 [M-H]$^-$	MS^2[593]: 285 (100)<br>MS^3[285] 257 (100), 267 (55), 241 (45), 213 (25)	594	Kaempferol 3-O-rutinoside [18]
8	18.04	461 [M-H]$^-$	MS^2[461]: 285 (100)<br>MS^3[285]: 257 (100), 267 (55), 229 (40), 213 (25), 197 (20)	462	Kaempferol 3-O-glucuronide [18]

Table S4. Qualitative LC-MS analysis of polyphenols in bean products.

Substance	RT [min]	Molecular Ion [m/z]	MS^n [m/z], rel. int. (%)	Molecular Weight [g/mol]	Tentative Identification
1	9.47	385 [M-H]$^-$	MS^2[385]: 191 (100)<br>MS^3[191]: 84 (100), 146 (30), 173 (10)	386	Feruloyl glucaric/galactaric acid derivative [23]
2	9.96	385 [M-H]$^-$	MS^2[385]: 191 (100)<br>MS^3[191]: 84 (100), 146 (30), 173 (10)	386	Feruloyl glucaric/galactaric acid derivative [23]
3	10.58	385 [M-H]$^-$	MS^2[385]: 191 (100)<br>MS^3[191]: 84 (100), 146 (30), 173 (10)	386	Feruloyl glucaric/galactaric acid derivative [23]
4	11.14	385 [M-H]$^-$	MS^2[385]: 191 (100)<br>MS^3[191]: 84 (100), 146 (30), 173 (10)	386	Feruloyl glucaric/galactaric acid derivative [23]
5	11.69	385 [M-H]$^-$	MS^2[385]: 191 (100)<br>MS^3[191]: 84 (100), 146 (30), 173 (10)	386	Feruloyl glucaric/galactaric acid derivative [23]

Table S5. Qualitative LC-MS analysis of polyphenols in soy products.

Substance	RT [min]	Molecular Ion [m/z]	MS^n [m/z], rel. int. (%)	Molecular Weight [g/mol]	Tentative Identification
1	12.9	417 [M+H]+	MS_2[417]: 255 (100)<br>MS^3[255]: 199 (100), 136 (70), 227 (55), 237 (30), 255 (15)	416	Daidzin [30]
2	13.3	447 [M+H]+	MS_2[447]: 285 (100)<br>MS^3[285]: 270 (100), 229 (20), 144 (10), 285 (5)	446	Glycitin [30]
3	14.3	433 [M+H]+	MS^2[433]: 271 (100)<br>MS^3[271]: 152 (100), 215 (80), 243 (70), 253 (40), 271 (10)	432	Genistin [30]
4	16.1	503 [M+H]+	MS_2[503]: 255 (100)<br>MS^3[255]: 199 (100), 136 (70), 237 (30), 255 (15)	502	Malonyl daidzin [30]
5	16.8	533 [M+H]+	MS_2[533]: 271 (100), 285 (60)	532	Malonyl glycitin [30]

			MS^3[271]: 215 (100), 152 (70), 243 (45), 253 (30), 271 (10)		
6	17.9	519 [M+H]+	MS_2[519]: 271 (100)<br>MS^3[271]: 215 (100), 152 (50), 243 (35), 253 (25), 271 (10)	518	Malonyl genistin [30]
7	18.4	255 [M+H]+	MS_2[255]: 199 (100), 136 (70), 227 (55), 237 (30), 255 (15)<br>MS^3[199]: 191 (100), 171 (30), 153 (15)	254	Daidzein [30]
8	20.5	271 [M+H]+	MS_2[271]: 152 (100), 215 (75), 243 (65), 253 (55), 271 (10)<br>MS^3[152]: 153 (100), 110 (45), 66 (40)	270	Genistein [30]

[18] Abu-Reidah, I. M.; Arráez-Román, D.; Lozano-Sánchez, J.; Segura-Carretero, A.; Fernández-Gutiérrez, A. Phytochemical Characterisation of Green Beans (Phaseolus Vulgaris L.) by Using High-Performance Liquid Chromatography Coupled with Time-of-Flight Mass Spectrometry. *Phytochem Anal* **2013**, *24* (2), 105–116. https://doi.org/10.1002/pca.2385.

[23] Nguyen, T.-K.-O.; Jamali, A.; Grand, E.; Morreel, K.; Marcelo, P.; Gontier, E.; Dauwe, R. Phenylpropanoid Profiling Reveals a Class of Hydroxycinnamoyl Glucaric Acid Conjugates in Isatis Tinctoria Leaves. *Phytochemistry* **2017**, *144*, 127–140. https://doi.org/10.1016/j.phytochem.2017.09.007.

[30] Lee, M. J.; Chung, I.-M.; Kim, H.; Jung, M. Y. High Resolution LC–ESI-TOF-Mass Spectrometry Method for Fast Separation, Identification, and Quantification of 12 Isoflavones in Soybeans and Soybean Products. *Food Chemistry* **2015**, *176*, 254–262. https://doi.org/10.1016/j.foodchem.2014.12.073.
